# Sintilimab Plus Apatinib and Chemotherapy as Second‑/Third-Line treatment for Advanced Gastric or Gastroesophageal Junction Adenocarcinoma: a prospective, Single-Arm, phase II trial

**DOI:** 10.1186/s12885-023-10661-4

**Published:** 2023-03-05

**Authors:** Le Zhang, Weixue Wang, Shaohua Ge, Hongli Li, Ming Bai, Jingjing Duan, Yuchong Yang, Tao Ning, Rui Liu, Xia Wang, Zhi Ji, Feixue Wang, Haiyang Zhang, Yi Ba, Ting Deng

**Affiliations:** Department of GI Medical Oncology, Key Laboratory of Cancer Prevention and Therapy, Tianjin’s Clinical Research Center for Cancer, Tianjin Medical University Cancer Institute and Hospital, National Clinical Research Center for Cancer, 300060 Tianjin, China

**Keywords:** Immune checkpoint inhibitors, Molecular targeted therapy, Sintilimab, Apatinib, Gastric cancer

## Abstract

**Background:**

The prognosis of patients with previously treated advanced gastric or gastroesophageal junction (GEJ) cancer remains poor. Given the robust development of immunotherapy and targeted therapy during the last decades, we aimed to investigate if the combination of traditional second-line chemotherapy with sintilimab and apatinib could bring survival benefits for these patients.

**Methods:**

In this single-center, single-arm, phase II trial, patients with previously treated advanced gastric or GEJ adenocarcinoma received specific dose level of intravenous paclitaxel or irinotecan (investigator’s choice), 200 mg intravenous sintilimab on day 1, and 250 mg oral apatinib once daily continuously in each cycle until disease progression, intolerable toxicity, or withdrawal of consent. The primary endpoints were objective response rate and progression-free survival. The secondary endpoints were mainly overall survival and safety.

**Results:**

From May 2019 to May 2021, 30 patients were enrolled. At the data cutoff date (March 19, 2022), the median follow-up duration was 12.3 months and 53.6% (95% CI, 33.9–72.5%) patients achieved objective response. The median progression-free survival and overall survival were 8.5 months (95% CI, 5.4–11.5) and 12.5 months (95% CI, 3.7–21.3), respectively. Grade 3–4 adverse events included hematological toxicities, elevated alanine aminotransferase, elevated aspartate aminotransferase, elevated alkaline phosphatase, elevated gamma-glutamyl transpeptidase, hyperbilirubinemia and proteinuria. The most frequent grade 3–4 adverse event was neutropenia (13.3%). No serious treatment-related adverse events or treatment-related deaths occurred.

**Conclusion:**

Sintilimab plus apatinib and chemotherapy demonstrates promising anti-tumor activity with manageable safety profile in patients with previously treated advanced gastric or GEJ cancer.

**Trial registration:**

ClinicalTrials.gov: NCT05025033, 27/08/2021.

## Background

Gastric cancer (GC) has become the sixth most common malignancy and the third leading cause of cancer-related death globally [1]. However, factors such as dietary preference and chronic *Helicobacter pylori* infection, make the condition even worse in East Asia, especially China [2]. For patients with advanced or recurrent metastatic GC, the prognosis remains poor [3–6].

For the second-line chemotherapy drugs recommended in the guideline, paclitaxel or irinotecan, the objective response rate (ORR) is only about 10–20.9% with a median progression-free survival (PFS) of 2.1 to 3.6 months, and a median overall survival (OS) of 5.3 to 9.5 months [7–10]. Efficacy of second-line paclitaxel plus ramucirumab [11], or other recommended subsequent regimens, including ramucirumab alone [12], nivolumab [13] or pembrolizumab alone [14], are also unsatisfactory. It is urgent to develop more effective options for gastric cancer patients in the second-line or subsequent treatment.

Recent studies suggested that anti-angiogenic therapeutic strategy such as ramucirumab or apatinib in combination with chemotherapy could achieve favorable results [11, 15, 16]. Ramucirumab is a human IgG1 monoclonal antibody against vascular endothelial growth factor receptor 2 (VEGFR-2), which was given intravenously. Apatinib is an oral small-molecule tyrosine kinase inhibitor, which can bind to and inhibit VEGFR-2 and was approved in China for the treatment of advanced GC after failure of second-line chemotherapy [17].

Increasing evidence of clinical studies support the use of immune checkpoint inhibitors (ICIs) in the treatment of GC, especially for patients with mismatch repair deficiency or microsatellite instability–high cancer [18–20]. The ATTRACTION-2 study showed that nivolumab produced significantly better outcomes than placebo as third- or later-line treatment of GC [13, 21]. The KEYNOTE-059 study demonstrated the efficacy of pembrolizumab in the third-line treatment of GC, especially in programmed cell death-ligand 1 (PD-L1) positive patients [14]. Sintilimab is a highly selective monoclonal IgG4 antibody that inhibits interactions between programmed cell death-1 (PD-1) and its ligands, and it also exerts a strong anti-tumor activity [22]. Studies have demonstrated the tolerance and pharmacological activity of sintilimab in patients with advanced GC and other solid tumors [23, 24].

Although immunotherapy has showed a partial curative effect on GC, the above studies showed that the efficacy of single-agent PD-1 monoclonal antibody was limited. Many studies have revealed a synergistic effect of PD-1/PD-L1 antibody plus chemotherapy or VEGFR2 inhibitor [23, 25–32]. To explore the mode and effect of combined treatment strategy as second-line and subsequent treatment of GC, we performed the present study to evaluate the efficacy and safety of chemotherapy combined with a PD-1 antibody (sintilimab) and VEGFR2 inhibitor (apatinib).

## Methods

### Study design and patients

This was a single-center, single-arm, phase II trial. Patients were enrolled from May 2019 to May 2021 at Tianjin Medical University Cancer Institute and Hospital in this study. The key inclusion criteria were:

age of ≥ 18 years;

Eastern Cooperative Oncology Group (ECOG) performance status of 0–2;

histologically confirmed metastatic or recurrent gastric or gastroesophageal junction (GEJ) adenocarcinoma;

previous first-line use of platinum combined with fluorouracil or disease progression within 6 months after withdrawal of adjuvant chemotherapy with platinum and fluorouracil-based regimen, or failure of second-line irinotecan or paclitaxel;

measurable lesions according to the Response Evaluation Criteria in Solid Tumors (RECIST) version 1.1;

adequate organ and bone marrow function (hemoglobin: ≥90 g/L, neutrophils: ≥1.5 × 10^9^/L, platelets: ≥100 × 10^9^/L, bilirubin: ≤1.5 × upper limit of normal [ULN], alanine aminotransferase [ALT] and aspartate aminotransferase [AST]: ≤2.5 × ULN [if liver metastasis is present, then ALT and AST ≤ 5 × ULN], endogenous creatinine clearance rate: ≥50 mL/min [Cockcroft–Gault formula], routine urinalysis: normal results or urine protein less than (++) or 24-hour protein urine of < 1.0 g, and normal blood coagulation with no active bleeding or thrombosis [international normalized ratio of ≤ 1.5 and activated partial thromboplastin time of ≤ 1.5 × ULN]);

estimated survival time of ≥ 3 months.

The key exclusion criteria included:

previous use of PD-1/PD-L1 inhibitor or other anti-angiogenesis targeted drug;

hypertension that could not be reduced to normal range by antihypertensive drugs;

clearly gastrointestinal bleeding tendency, including locally active ulcer lesions, fecal occult blood (++), or a history of melena and hematemesis within 1 month;

serious cardiovascular and cerebrovascular diseases;

immune system diseases requiring treatment;

inability to swallow or intestinal obstruction;

contraindication or allergy to the study drugs;

concomitant diseases that seriously endanger the safety of the patient or affect the completion of the study.

The study was approved by the independent ethics committees of Tianjin Medical University Cancer Institute and Hospital and was performed in accordance with the Declaration of Helsinki. All enrolled patients provided written informed consent. This trial was registered with ClinicalTrials.gov, number NCT05025033.

### Treatment and assessments

Apatinib was given as a fixed dose of 250 mg once a day, within 30 min after breakfast. Sintilimab was given within 60 min before chemotherapy by intravenous infusion of a fixed dose of 200 mg every 3 weeks. Antiemetic drugs were given by intravenous infusion 30 min before chemotherapy with either irinotecan (150 mg/m^2^, 90-minute intravenous infusion once every 2 weeks) or paclitaxel (150 mg/m^2^, 3-hour intravenous infusion once every 3 weeks). Considering that compared with monotherapy combination may increase the risk of toxic reactions, and chemotherapy is more important to play an immunomodulatory role in the combination therapy, the dose of chemotherapy drugs in this study was lowered compared with the conventional dose. Prophylactic atropine was given before the next irinotecan infusion treatment for patients with acute cholinergic syndrome. To prevent allergic reaction, 10 mg of oral dexamethasone was given 12 and 6 h before paclitaxel administration, and 400 mg of intravenous cimetidine and 50 mg of intramuscular diphenhydramine were given 30 min before paclitaxel administration. An intravenous infusion of sintilimab every 3 weeks is considered one cycle. Apatinib and sintilimab were continued until the disease progressed or became intolerable or up to 2 years, and the chemotherapy drug irinotecan or paclitaxel was continued until the disease progression, intolerable toxicity, or up to 6 months. If adverse events occurred during treatment, symptomatic and supportive treatments were given according to clinical protocol. Immune-related adverse events were treated with reference to the NCCN immune-related toxicity management guidelines. Blood or urine laboratory tests such as routine blood parameters, blood biochemistry, heart function, routine urinalysis, and thyroid function were regularly performed according to clinical protocol. Contrast-enhanced computed tomography of chest, abdomen, and pelvis was carried out every 6 weeks until progressive disease (PD) was confirmed. Response was determined by the investigators according to RECIST 1.1. Adverse events were assessed according to the National Cancer Institute Common Terminology Criteria for Adverse Events (CTCAE) version 5.0. Patients with PD after the study treatment were followed up every 3 months by telephone until death.

### Endpoints

The primary endpoints were ORR and PFS. Secondary endpoints were OS, disease control rate (DCR), duration of response (DOR), and safety. ORR was defined as the percentage of patients who had a complete response (CR) or partial response (PR). DCR was defined as the percentage of patients who had a CR, PR, or stable disease (SD). DOR was defined as the time from the first assessment of a tumor as CR or PR to PD or any-cause death. PFS was defined as the time from the date of enrollment to progression or any-cause death. OS was defined as the time from the date of enrollment to any-cause death. The date of last follow-up was recorded as censored data for survival analysis when the time of death or progression could not be confirmed or if the patient was still alive.

### Statistical analysis

Using the Simon’s optimal two-stage design, with the power of 80% and one-sided α of 5%, the planned sample size was 22 patients. Considering the efficacy of anti-PD-1 monoclonal antibody combined with anti-angiogenesis targeted therapy, we set the primary endpoint PFS at 5 months, and we expected that the ORR could increase from 10 to 35%. In the first stage, we enrolled 8 patients. If 1 or more patients reached an objective response, we would enroll additional 14 or more patients in the second stage. Ultimately, if 5 or more of 22 patients reached an objective response, the primary endpoint was met.

Patients who completed at least one treatment cycle with at least one follow-up tumor assessment were considered evaluable for efficacy. All patients who had undergone at least one treatment cycle were included in the safety assessment. The 95% CIs of ORR and DCR were calculated using exact binomial test. Median PFS, OS and their 95% CIs, were estimated using the Kaplan–Meier method and compared using log-rank test. Hazard ratio (HR) and 95% CIs were calculated using Cox proportional hazard model. Exploratory analyses were performed between subgroups by sex, primary tumor site, ECOG performance status, pathological grade, Lauren classification, metastasis site, and liver metastasis. All statistical analyses were two-sided and significance was set at *P* < 0.05. The software used for all statistical analyses was SPSS Statistics (version 22.0).

## Results

### Patient characteristics

In the first stage, 2 in 8 patients had a PR, and 22 patients were enrolled in the second stage. The baseline characteristics of 30 patients are summarized in Table 1.

**Table 1:** Demographics and baseline characteristics

Characteristic	Patients, n (%)
Age, years, median (range)	59(28–73)
Sex	
Male Female	23(76.7) 7(23.3)
ECOG PS*	
0 1 2	4(13.3) 25(83.3) 1(3.3)
Primary tumor sites	
Lower esophagus Cardiac and gastric fundus Gastric body Gastric antrum	1(3.3) 7(23.3) 13(43.3) 9(30.0)
Cancer grading	
G1 G2 G3	0(0) 5(16.7) 25(83.3)
Lauren typing	
Intestinal type Diffuse type Mixed type or unknown	9(30.0) 14(46.7) 7(23.3)
Metastatic sites	
Lymph node Peritoneum Liver Bone Lung	19(63.3) 9(30.0) 8(26.7) 2(6.7) 1(3.3)
Prior treatment regimens	
First-line Second-line Platinum and Fluorouracil Taxanes	25(83.3) 5(16.7) 29(96.7) 7(23.3)
MMR/MSI*	
MMR deficient MMR proficient	0(0) 30(100.0)
HER2*	
0 1+ 2+/FISH*(-)	22(73.3) 4(13.3) 4(13.3)
EBER*	
Negative Positive	30(100.0) 0(0)

*ECOG PS: Eastern Cooperative Oncology Group Performance Status

MMR: Mismatch Repair

MSI: Microsatellite Instability

HER2: Human Epidermal Growth Factor Receptor 2

FISH: Fluorescence in Situ Hybridization

EBER: Epstein-Barr virus-encoded RNA

**Fig. 1:**
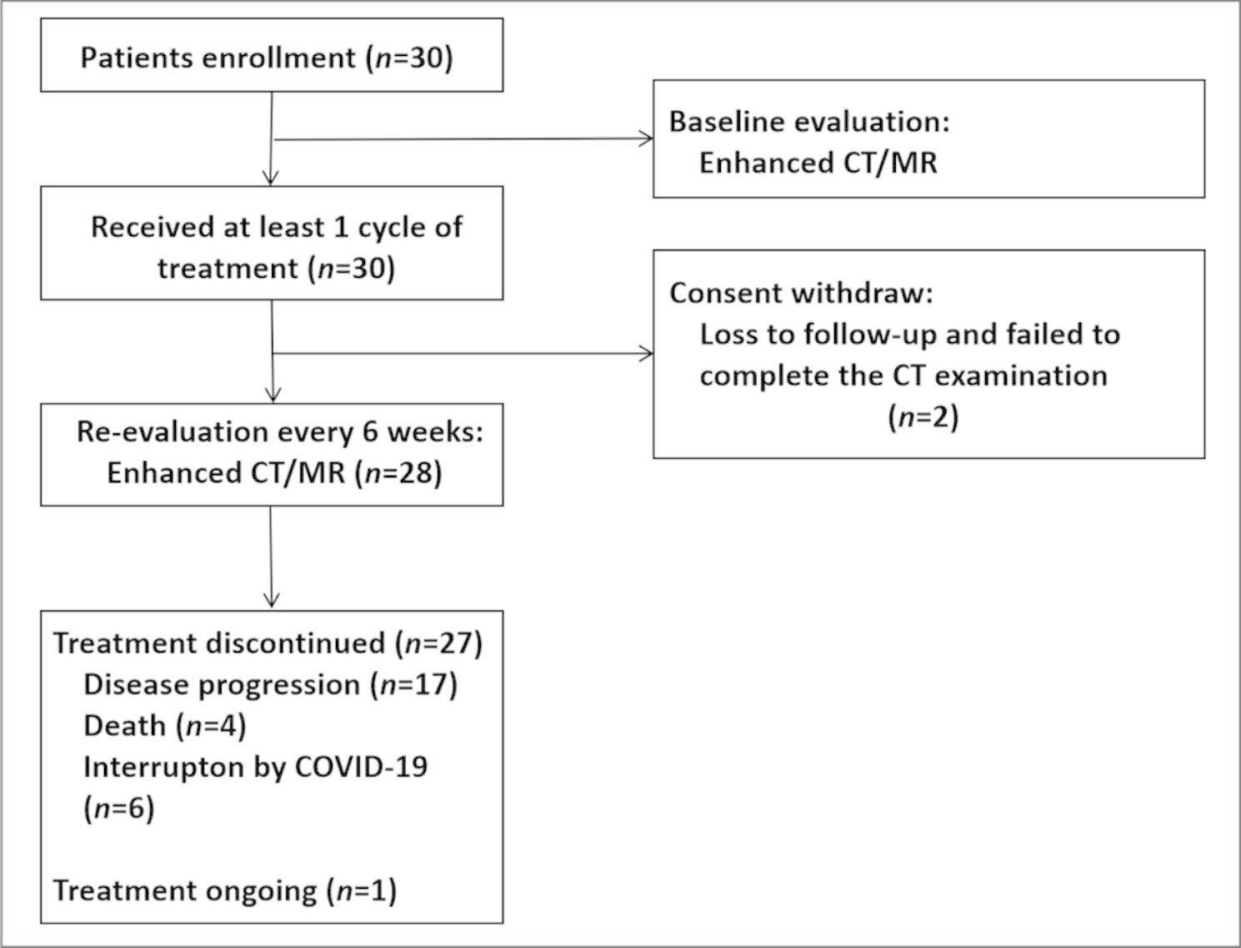
The flowchart of the study

At the data cutoff (March 19, 2022), the median follow-up duration was 12.3 months. 21 patients discontinued this protocol treatment due to disease progression or death, 2 patients due to consent withdraw, and 6 patients dropped out of the study after treatment was interrupted by the COVID-19 for more than 6 weeks. The flowchart of the study is shown in Fig. 1.

### Efficacy

**Fig. 2:**
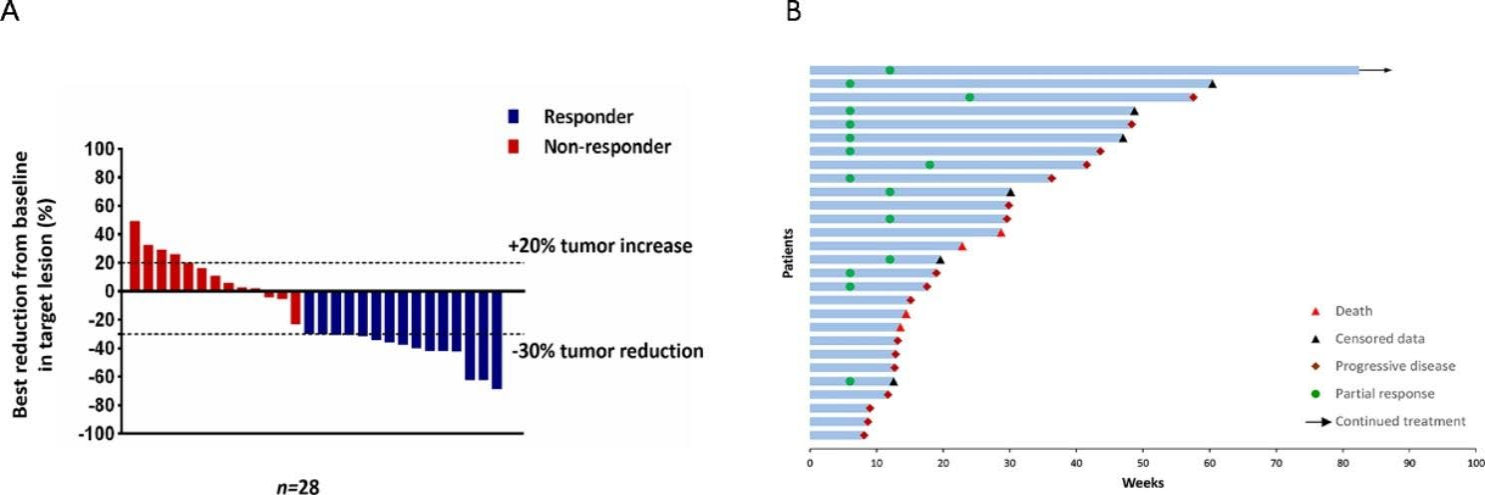
Waterfall plot of the best percentage change from baseline in measurable tumor lesions of 28 measurable patients (A) and duration of response (*n* = 28) (B)

**Fig. 3:**
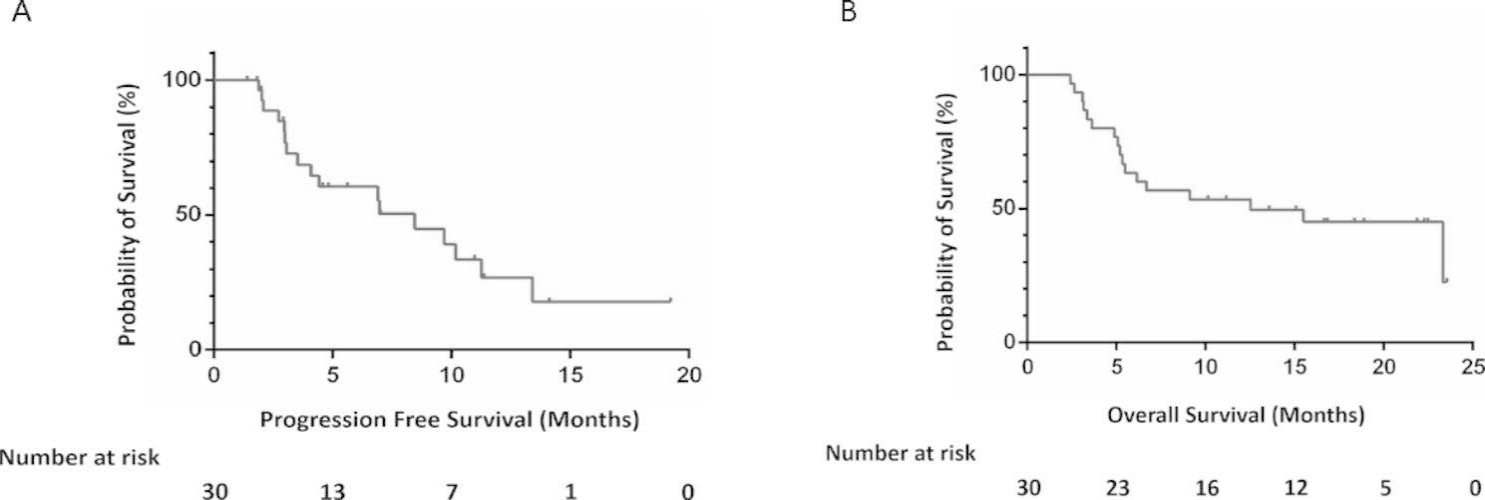
Kaplan-Meier survival curves of progression-free survival (PFS) (A) and overall survival (OS) (B) in full analysis set of the 30 patients

Of 30 patients, 2 could not be evaluated because they did not complete the computed tomography examination after undergoing one treatment cycle. Among 28 patients with evaluable efficacy, PR was obtained in 15, SD in 8, and PD in 5. The ORR was 53.6% (95% CI, 33.9–72.5%), and the DCR was 82.1% (95% CI, 63.1–93.9%) (Table 2). The best percentage change from baseline in measurable tumor lesions and characteristics of objective response in 28 evaluable patients are shown in Fig. 2. The median DOR was 8.8 months (95% CI, 6.0-11.5) (Table 2). The median PFS was 8.5 months (95% CI, 5.4–11.5), and the median OS was 12.5 months (95% CI, 3.7–21.3) (Fig. 3).

**Table 2:** Summary of tumor response and survival data

Best overall response	Patients
CR*, n (%)	0(0)
PR*, n (%)	15(50.0)
SD*, n (%)	8(26.7)
PD*, n (%)	5(16.7)
NE*, n (%)	2(6.7)
ORR*, n (%)	15/28(53.6)
DCR*, n (%)	23/28(82.1)
DOR* (months), median (95% CI*)	8.8(6.0-11.5)
PFS* (months), median (95% CI)	8.5(5.4–11.5)
OS* (months), median (95% CI)	12.5(3.7–21.3)

*CR: Complete response

PR: Partial response

SD: Stable disease

PD: Progressive disease

NE: Not evaluable

ORR: Objective response rate

DCR: Disease control rate

DOR: Duration of response

PFS: Progression Free Survival

OS: Overall Survival

CI: Confidence interval

According to the best percentage change from baseline in measurable tumor lesions, responder was defined as the patient who had a complete response (CR) or partial response (PR), while non-responder was defined as the patient who had a stable disease (SD) or progressive disease (PD).

### Survival correlation analysis

Sex, primary tumor site, ECOG performance status, pathological grade, Lauren classification, metastasis site, liver metastasis, previous treatment regimen, and number of chemotherapy lines were all included in the Cox univariate analysis for PFS and OS (Table 3). The results showed significant differences in both PFS (HR: 0.187, 95% CI, 0.042–0.840, *P* = 0.029) and OS (HR: 0.214, 95% CI, 0.049–0.947, *P* = 0.042) in subgroup by liver metastasis, as well as PFS in subgroup by Lauren classification (HR: 0.297, 95% CI, 0.095–0.926, *P* = 0.036). Liver metastasis and Lauren classification were therefore included in the multivariate analysis. The results showed that liver metastasis was an independent prognostic factor for PFS (HR: 0.208, 95% CI, 0.044–0.988, *P* = 0.048), whereas the Lauren classification did not reach statistical significance (HR: 0.322, 95% CI, 0.087–1.188, *P* = 0.089). Similarly, liver metastasis was also an independent prognostic factor for OS (HR: 0.214, 95% CI, 0.049–0.947, *P* = 0.042).

**Table 3:** Cox univariate analysis of PFS and OS.

	PFS				OS		
	HR*	**95% CI**	*P* **value**		**HR**	**95% CI**	*P* **value**
Sex	0.676	0.236–1.932	0.465		1.572	0.447–5.520	0.481
Age	0.943	0.360–2.469	0.904		0.699	0.265–1.842	0.469
Primary tumor sites	1.163	0.401–3.373	0.782		0.874	0.281–2.716	0.816
ECOG PS	0.885	0.285–2.752	0.833		1.080	0.306–3.816	0.905
Cancer grading	0.396	0.090–1.739	0.220		1.090	0.349–3.403	0.882
Lauren typing	0.297	0.095–0.926	0.036		0.529	0.169–1.656	0.274
Liver Metastasis	0.187	0.042–0.840	0.029		0.214	0.049–0.947	0.042
Metastasis site	0.430	0.161–1.151	0.093		0.595	0.220–1.606	0.306
Previous regimen	0.046	0-15955.418	0.636		2.688	0.340-21.246	0.348
Chemotherapy lines	1.123	0.318–3.968	0.858		1.213	0.345–4.268	0.764

*HR: hazard ratio

**Fig. 4:**
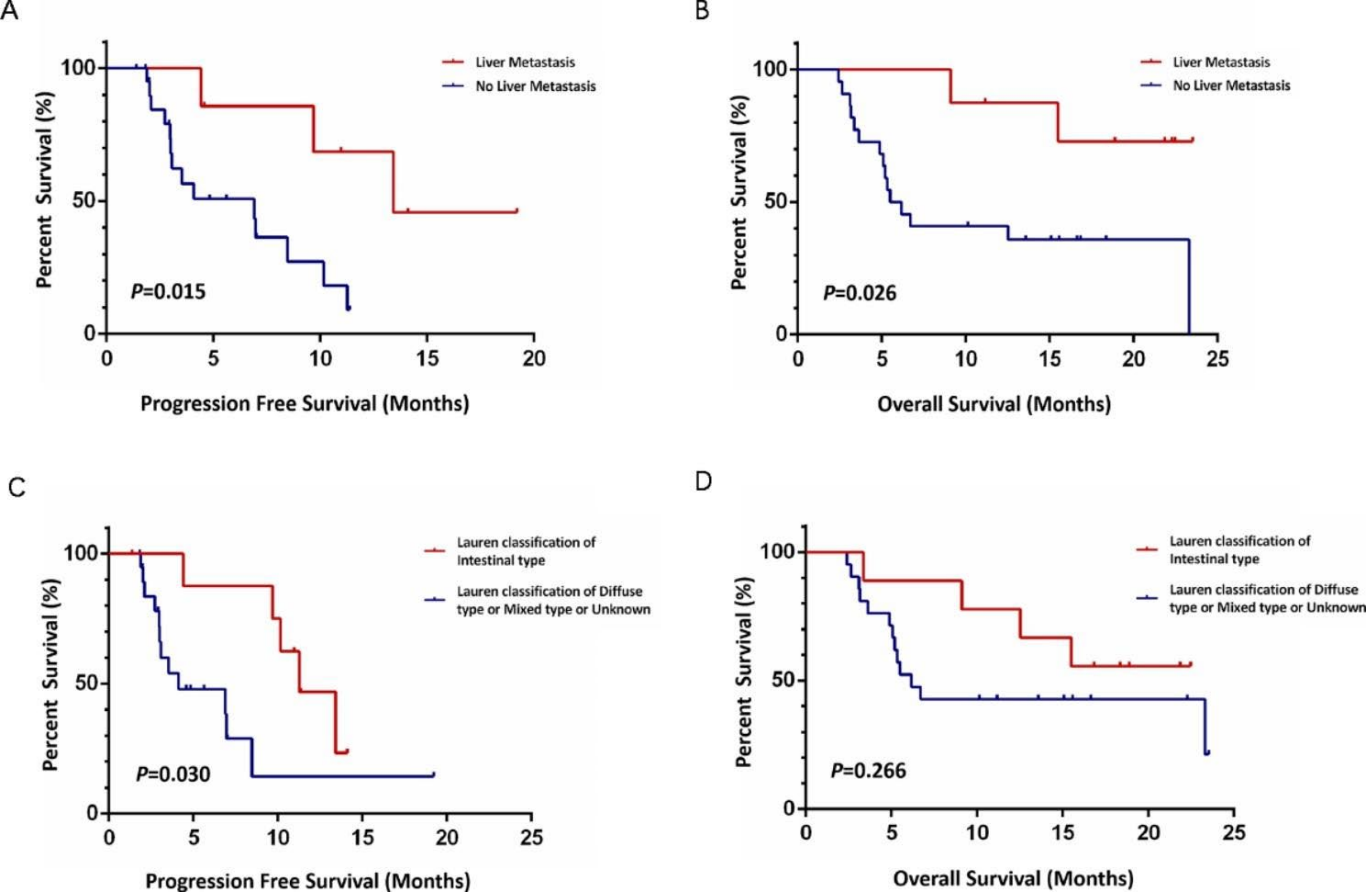
Kaplan–Meier curves for PFS and OS of subgroup analysis. PFS (A) and OS (B) curves comparing the two groups with liver metastatic or not; PFS (C) and OS (D) curves comparing the two groups with Lauren Classification of intestinal type or other types

Eight patients had liver metastasis, among whom 7 could be assessed; all 7 of these patients attained PR. Twenty-two patients had non-liver metastasis, among whom 21 could be assessed: 8 attained PR, 8 achieved SD, and 5 developed PD. The ORR in patients with liver metastasis and non-liver metastasis was 100% and 38.1%, respectively (*P* = 0.007), and the median PFS was 13.4 and 6.9 months, respectively (*P* = 0.015) (Fig. 4A). Among patients with liver metastasis, 6 patients were still alive, and the median OS was not reached; the median OS time in patients with non-liver metastasis was 5.5 months. There was a significant difference in OS between the two groups (*P* = 0.026) (Fig. 4B).

Nine patients had intestinal type tumors by the Lauren classification; of these, 8 achieved PR and one attained SD. Twenty-one patients had diffuse, mixed, or unknown tumors. Nineteen of these 21 patients were able to be assessed: 7 achieved PR, 7 attained SD, and 5 developed PD. The ORR of intestinal type tumors was 88.9%, and that of diffuse/mixed/unknown tumors was 36.8% (*P* = 0.016). The median PFS in patients with intestinal type tumors was 11.3 months, and that of patients with diffuse/mixed/unknown tumors was 4.1 months (*P* = 0.030) (Fig. 4C). The median OS was not reached in patients with intestinal type tumors, and that in patients with diffuse or mixed type tumors was 6.2 months (*P* = 0.266) (Fig. 4D). No significant difference between 2nd and 3rd line of treatment or chemotherapy of paclitaxel and irinotecan, but it can be seen that OS of second-line treatment showed a longer trend than third-line treatment (15.5 months vs. 9.1 months, *P* = 0.764), and the survival of patients treated with irinotecan chemotherapy also showed a longer trend than those treated with paclitaxel (23.3 months vs. 6.7 months, *P* = 0.275).

### Safety

The most common adverse events were leukopenia (56.7%), anemia (56.7%), neutropenia (56.7%), alopecia (53.3%), fatigue (46.7%), elevated AST (40.0%), anorexia (36.7%), elevated ALT (36.7%), hypothyroidism (36.7%), proteinuria (36.7%) and nausea (30.0%). However, most of these adverse events were grade 1 or 2. The most common grade 3 or 4 treatment-related adverse event was neutropenia (13.3%), followed by leukopenia (6.7%). Two patients developed grade ≥ 3 neutropenia with fever and recovered after supportive treatment. One patient developed grade 3 anemia and recovered after a blood transfusion. One patient developed grade 3 hepatic transaminase elevation, including ALT, AST, alkaline phosphatase, and gamma-glutamyl transpeptidase. This was considered as an immune-related adverse event and was resolved by glucocorticoid treatment. One patient developed grade 3 bilirubin elevation, which resolved after treatment with a glucocorticoid combined with mycophenolate mofetil, considering immune-related factors. No treatment-related deaths occurred. Details of adverse events are shown in Table 4.

**Table 4:** Treatment-Related Adverse Events

Terms	All grades, n (%)	Grade1/2, n (%)	Grade3/4, n (%)
Leukopenia	17(56.7)	15(50.0)	2(6.7)
Neutropenia	17(56.7)	13(43.3)	4(13.3)
Febrile neutropenia	2(6.7)	-	2(6.7)
Thrombocytopenia	8(26.7)	8(26.7)	0(0)
Anemia	17(56.7)	16(53.3)	1(3.3)
Fatigue	14(46.7)	14(46.7)	0(0)
Anorexia	11(36.7)	11(36.7)	0(0)
Nausea	9(30.0)	9(30.0)	0(0)
Emesis	8(26.7)	8(26.7)	0(0)
Alopecia	16(53.3)	16(53.3)	0(0)
Diarrhea	5(16.7)	5(16.7)	0(0)
Elevated alanine aminotransferase	11(36.7)	10(33.3)	1(3.3)
Elevated aspartate aminotransferase	12(40.0)	11(36.7)	1(3.3)
Elevated alkaline phosphatase	9(30.0)	8(26.7)	1(3.3)
Elevated gamma-glutamyl transpeptidase	8(26.7)	7(23.3)	1(3.3)
Hyperbilirubinemia	3(10.0)	2(6.7)	1(3.3)
Hyperglycemia	6(20.0)	6(20.0)	0(0)
Hyponatremia	6(20.0)	6(20.0)	0(0)
Hypokalemia	7(23.3)	7(23.3)	0(0)
Hypermagnesemia	7(23.3)	7(23.3)	0(0)
Hyperphosphatemia	2(6.7)	2(6.7)	0(0)
Hypercalcemia	1(3.3)	1(3.3)	0(0)
Hypocalcemia	7(23.3)	7(23.3)	0(0)
Hypothyroidism	11(36.7)	11(36.7)	0(0)
Proteinuria	11(36.7)	10(33.3)	1(3.3)
Hypertension	5(16.7)	5(16.7)	0(0)
Rash	1(3.3)	1(3.3)	0(0)
Immune pneumonia	1(3.3)	1(3.3)	0(0)
Cholinergic syndrome	2(6.7)	2(6.7)	0(0)

## Discussion

This study explored the combination of sintilimab plus apatinib and chemotherapy. With an ORR reaching 53.6% and a long median PFS (8.5 months), this prospective trial met the primary endpoint. The median OS time was > 1 year, reaching 12.5 months. These results indicate a significant improvement over previous second-line treatments, suggesting that the combination of a PD-1 inhibitor, VEGFR inhibitor, and chemotherapy has a certain synergistic effect.

For the first-line treatment of advanced GC, the anti-PD-1 combination therapy has been listed in the latest treatment guidelines. The CheckMate 649 study showed that nivolumab combined with chemotherapy improved the OS compared with chemotherapy alone [33]. Sintilimab plus chemotherapy provided a new standard first-line treatment option for Chinese patients with gastric or GEJ cancer, demonstrating superior OS and PFS with an acceptable safety profile according to the ORIENT-16 study [34]. However, there are relatively few studies on second-line therapy for advanced GC.

Previous studies have also shown a synergistic effect between chemotherapy and apatinib [35]. In a study by Xu et al. [16], apatinib improved the efficacy of paclitaxel and 5-fluorouracil both in vitro and in vivo. In addition, apatinib has immunomodulatory effects mediated by decreases in regulatory T cells and myeloid-derived suppressor cells as well as enhanced dendritic cell maturation and effector T-cell infiltration[27-30]. Liang et al. [31] found that the combination of anti-PD-1 antibody camrelizumab and apatinib for advanced solid tumors was more effective than immunotherapy alone (ORR, 34.2% vs. 18.8%; median PFS, 6.0 vs. 4.5 months, *P* = 0.002). Xu et al. [32] explored the safety and tolerability of apatinib at different doses (125–500 mg) in combination with camrelizumab in patients with advanced GC and advanced liver cancer. The phase Ia-identified recommended phase II dose was 250 mg, and 250 mg of apatinib combined with 200 mg of camrelizumab was used for a expansion study. The results showed that safety was controllable. On this basis, we conducted this exploratory study of anti-PD-1 antibody plus apatinib and chemotherapy in patients with advanced GC.

Our study of the combination of sintilimab plus apatinib and chemotherapy (irinotecan or paclitaxel) in an unselected population showed an ORR of 53.6%, median PFS of 8.5 months, and median OS of 12.5 months. These results are better than those of ramucirumab combined with paclitaxel in the RAINBOW study (ORR of 28%, median PFS of 4.4 months, and median OS of 9.6 months) [11], paclitaxel or pembrolizumab in the KEYNOTE-061 study (ORR of 16% vs. 14%, median PFS of 1.5 vs. 4.1 months, and median OS of 9.1 vs. 8.3 months) [36], and apatinib combined with camrelizumab (ORR of 17.4%, median PFS of 2.9 months, and median OS of 11.4 months) [32]. In a phase I/II study of nivolumab combined with paclitaxel plus ramucirumab as second-line treatment in 43 patients with advanced GC, the ORR was 37.2%, the median PFS was 5.1 months, and the median OS was 13.1 months [37]. The results are similar to our study, suggesting that the combination of the three drugs is a treatment option worthy of further verification and has promising anti-tumor activity.

Although recent studies have indicated that PD-L1 levels are related to the efficacy of PD-1 inhibitors, the CheckMate 649 study showed that nivolumab combined with chemotherapy improved the OS, especially in patients with a Combined Positive Score (CPS) of ≥ 5[33]. In the unselected population from the ATTRACTION-4 study, nivolumab combined with chemotherapy failed to improve OS compared with placebo combined with chemotherapy, and advantages only existed in terms of the ORR and PFS [38]. In addition, pembrolizumab plus chemotherapy in the KEYNOTE-062 study did not result in a significant improvement in efficacy, regardless of the CPS [39]. Therefore, CPS is not the only predictor of the efficacy of PD-1 antibody, especially in the mode of combination therapy. Other relevant influencing factors need to be further explored.

The univariate Cox regression analysis of this study showed that the Lauren classification and liver metastasis were potential prognostic factors affecting both PFS and OS, whereas the multivariate analysis showed that liver metastasis was an independent prognostic factor for PFS and OS. Patients with liver metastasis had significantly better PFS and OS and significantly better ORR than those with non-liver metastasis. This result suggests that patients with liver metastasis are more likely to benefit from the treatment regimen in this study. However, Tumeh et al. [40] found that patients with liver metastasis of melanoma or non-small cell lung cancer who were treated with pembrolizumab showed reduced response and PFS and that liver metastasis was associated with reduced marginal CD8 + T-cell infiltration, providing a potential mechanism for the worse prognosis. Akinori et al. [41] also found that liver metastasis, ECOG performance status of 1 or 2, and a large sum of target lesion diameters at baseline were significantly associated with hyper-progressive disease in 62 patients with advanced GC treated with nivolumab. The results of the two above-mentioned studies are seem to be contradictory to those in our studies. The reasons for this discrepancy may be as follows. First, there may have been differences for the prognosis of hepatic metastasis among different tumor types. Furthermore, the regimen in this study involved immunotherapy combined with anti-angiogenesis and chemotherapy, but the regimens in the above two studies involved immunotherapy monotherapy. The combination of three drugs may change the treatment outcome of liver metastasis. However, because of the small number of patients, the conclusions of this study need to be verified by expanding the sample size.

The ORR obtained by patients with intestinal type tumors according to the Lauren classification was significantly better than that of patients with diffuse type, mixed type, or unknown tumors, and there was also a significant difference in the median PFS (11.3 vs. 4.1 months, *P* = 0.030). Although the median OS was prolonged, the difference did not reach statistical significance (not reached vs. 6.2 months, *P* = 0.266). However, a retrospective study by Nie et al. [24] showed that the efficacy of sintilimab in patients with intestinal subtype GC (ORR, 30.0%; DCR, 80.0%) was superior to that in patients with non-intestinal subtype GC (ORR, 6.3%; DCR, 56.3%); additionally, patients with the intestinal subtype obtained a longer PFS (4.0 vs. 1.9 months) and OS (9.0 vs. 4.1 months) than those with the non-intestinal subtype. These findings are basically consistent with the conclusions of our study, suggesting that patients with the intestinal type of GC according to the Lauren classification are more likely to benefit from immunotherapy. However, because of the small number of patients in the above studies and the present study, this conclusion requires further verification.

No unexpected adverse events occurred in this study. Most of the adverse events were grade 1 and 2. The most common grade 3–4 treatment-related adverse event was neutropenia, which was mainly caused by irinotecan. The most common adverse event caused by sintilimab was increased liver transaminases, followed by hypothyroidism; most of these cases were grade 1 and 2. The most common adverse events related to apatinib were proteinuria and hypertension, most of which were also grade 1 and 2. The overall tolerance of this treatment regimen was good, likely because of the reduced dose of chemotherapy drugs and apatinib used in this study compared with the standard dose when used as monotherapy, on account of that we thought they were more important to play an immunomodulatory role in the combination therapy.

This study has some limitations. Firstly, this was a single-arm, single-center study without a randomized control group; thus, it lacked sufficient persuasiveness. Secondly, patients enrolled in this study were HER-2-negative and had a proficient mismatch repair status; however, the lack of more molecular feature detections may bring some limits for further mechanism analysis, such as PD-L1 levels and so on. In addition, the number of patients was small, which may lead to some bias in the study results. A prospective expansion study of this regimen is underway.

## Conclusion

In summary, this study explored sintilimab plus apatinib and chemotherapy (paclitaxel or irinotecan) as a second-line or subsequent treatment regimen for advanced GC, showing promising efficacy. A higher ORR and longer PFS and OS were observed in patients with liver metastasis than in those with non-liver metastasis, and a higher ORR and longer PFS were observed in patients with intestinal type according to the Lauren classification. There was a trend of OS prolongation, and the regimen was well-tolerated and deserves further research.

## Data Availability

The datasets used and/or analyzed during the current study are available from the corresponding author upon reasonable request.

## List of Abbreviations

GEJ: Gastroesophageal junction
GC: Gastric cancer
ORR: Objective response rate
PFS: Progression-free survival
OS: Overall survival
VEGFR2: Vascular endothelial growth factor receptor 2
ICIs: Immune checkpoint inhibitors
PD-L1: Programmed cell death-ligand 1
PD-1: Programmed cell death-1
ECOG: Eastern Cooperative Oncology Group
RECIST: Response Evaluation Criteria in Solid Tumors
PD: Progressive disease
CTCAE: Common Terminology Criteria for Adverse Events
DCR: Disease control rate
DOR: Duration of response
CR: Complete response
PR: Partial response
SD: Stable disease
HR: Hazard ratio
MMR: Mismatch Repair
MSI: Microsatellite Instability
HER2: Human Epidermal Growth Factor Receptor 2
FISH: Fluorescence in Situ Hybridization
EBER: Epstein-Barr virus-encoded RNA
